# Effects of Physical and Chemical States of Iron-Based Catalysts on Formation of Carbon-Encapsulated Iron Nanoparticles from Kraft Lignin

**DOI:** 10.3390/ma11010139

**Published:** 2018-01-15

**Authors:** Xuefeng Zhang, Qiangu Yan, Jinghao Li, Jilei Zhang, Zhiyong Cai

**Affiliations:** 1 Department of Sustainable Bioproducts, Mississippi State University, Mississippi State, MS 39762, USA; njfuxf@gmail.com; 2 U.S. Department of Agriculture, Forest Service, Forest Products Laboratory, Madison, WI 53726, USA; csuftljh@gmail.com

**Keywords:** kraft lignin, biopolymer, iron nanoparticles, iron nitrate, thermal treatment, carbon-encapsulated iron nanoparticles

## Abstract

Effects of physical and chemical states of iron-based catalysts on the formation of carbon-encapsulated iron nanoparticles (CEINs) synthesized thermally from kraft lignin were investigated. Experimental results indicated that if solution-based iron nitrate (FeN) was used as an iron source for the catalyst, CEINs observed were α-Fe and γ-Fe-based cores encapsulated by few layers graphitic-carbon (mostly 1–5 layers) and the majority of these CEINs were embedded in amorphous carbon matrix. The formation of graphitic-carbon shells is believed based on the dissolution and precipitation mechanism of amorphous carbon acting as the carbon source. If solid-based iron nanoparticles (FePs) were used as the catalyst, CEINs observed were α-Fe, γ-Fe, and Fe_3_C-based cores encapsulated with tangled graphitic-carbon nanoribbons and carbon tubules and the majority of these CEINs were found along the edge of amorphous carbon matrix. The growth of tangled graphitic-carbon nanoribbons and carbon tubules is based on a chemical vapor decomposition process, i.e., the carbonaceous gases from kraft lignin decomposition served as the carbon source.

## 1. Introduction

Lignin is the most abundant, highly cross-linked aromatic biopolymer on Earth, and mainly composes of carbon (~60%), oxygen (~30%), and hydrogen (~6%). More than 70 million tons of lignin is produced from wood pulping process every year as low-value by-products [1]. Most chemical pulping lignin (98%) is simply burned on-site for the recovery of energy and pulping chemicals, and kraft lignin (KL) accounts for 80% of these chemical pulping lignin [2]. Lignin contains more than 60% of carbon and can be potentially used for the production of value-added graphitic-carbon nanostructures like carbon-encapsulated iron nanoparticles (CEINs). 

CEINs draw much attention from worldwide research groups because of their unique electric and magnetic properties. With appropriate functionalization [3], CEINs can be used in various applications required severe temperature and humidity conditions such as data storage, catalyst, environmental remediation, and biomedical area because the carbon shell structure prevents oxidation of iron nanoparticles [4]. CEINs can be produced through denotation [5], laser irradiation [6], flame synthesis [7], arc discharge [8,9] and chemical vapor decomposition (CVD) methods [10]. CVD process is normally used for the production of graphitic-carbon nanostructures, where carbonaceous gases/vapors pass through tubular reactor heating zone (800 to 1200 °C) and react with iron-based catalysts to form graphitic-carbon nanostructures [10]. The CVD processes have its own advantages of high purity of as-synthesized CEINs, however, the high synthesis cost and the limitation of precursor (non-renewable carbon precursors are usually used), limited the process scalability [11].

In recent years, solid carbon precursors such as graphite powder, synthetic polymers, and lignocellulosic materials are thermally treated at 800 to 1200 °C [4,12,13,14,15,16] using transition metal particles and various these metal salts as catalysts. Scientists tend to believe that the formation of graphitic-carbon nanostructures on transition metal (for instance iron) particle surfaces is because solid carbon materials directly react with transition metal nanoparticles at high temperature through dissolution and precipitation mechanism [4,12,15]. The catalytic activity of these transition metals is affected by the metal physical (such as particle size, how well particles dispersed in carbon precursors) and chemical (such as valence number) states. For instance, in a typical catalytic graphitization process using solution-based metal salts as a catalyst, the first step is to transform metal salt to zero-valent metal nanoparticles, and follow by metal nanoparticles catalytically converting of carbon materials to graphitic-carbon nanostructures, and this process is different from the process where solid-based metal particles are used as a catalyst [12]. In addition, the dispersion quality of solid-based metal particles in polymer precursors cannot be the same as that of solution-based metal salts during the catalyst and carbon source material mixing because chemical bonds can be formed between metal ions (only for solution-based metal salts) and polymers through chelation [17]. 

Limited literature was found in investigating how physical and chemical states of iron-based catalysts affect the formation of CEINs synthesized from lignin as a carbon source [17], especially, KL. In this study, KL promoted with solid-based iron nanoparticles (FePs) and solution-based iron nitrate (FeN) as iron catalysts was thermally treated at 1000 °C. The dispersion of catalysts in lignin was studied using Fourier transform infrared spectroscopy (FTIR) and X-ray diffraction (XRD), the thermal decomposition behavior of lignin-catalyst mixture was investigated using thermal gravimetric (TG) analyses. The morphology and structure of as-synthesized CEINs were characterized and compared using XRD, scanning electron microscopy (SEM), and transmission electron microscopy (TEM). The as-synthesized CEINs can be used as an absorbent for environmental remediation.

## 2. Materials and Methods

### 2.1. Materials

Deionized-water purified softwood KL with its commercial name of Bio-Choice lignin was supplied by Domtar Corp. (Plymouth, NC, USA). The KL ash content measured in our lab was 0.53%, following ASTM D1102 [18]. FePs purchased from Sun Innovations Inc. (Fremont, CA, USA) averaged 25 nm in diameter. FeN (Fe(NO_3_)_3_·9H_2_O, 98% purity) was purchased from Sigma-Aldrich (St. Louis, MO, USA).

### 2.2. Precursors Preparation

For the experiment using solid-based FePs as the catalyst, the KL/FePs precursor was prepared through manually grounding the mixture of 0.5 g of FePs (1 part of Fe in weight) and 2 g of oven-dry KL (4 parts) solids in an agate mortar for five minutes. For the experiment using solution-based FeN as an iron source for the catalyst, the KL/FeN precursor was prepared through first mixing 18.32 g of FeN (1 part of Fe in weight) and 10 g of oven-dry KL (4 parts) solids in 200 mL of distilled water, secondly stirring the mixture in an oil-bath (120 °C) for 30 min, and finally drying the sticky mixture at 80 °C for 2 h then another 24 h at 105 °C.

### 2.3. Thermal Treatment

Thermal treatments of KL, KL/FePs and KL/FeN samples were carried out in a split-hinge quartz tube Lindberg/Blue M 1200 electric furnace (ThermoFisher Scientific, Waltham, MA USA) equipped with a Lindberg/Blue UTC 150 temperature controller (ThermoFisher Scientific, Waltham, MA, USA), respectively. The quartz tube had an inner diameter of 1.84 in. and a length of 32 in. For each experimental run, 2.5 g of samples were loaded into each of two boats placed in the middle of quartz tube. The thermal treatment process started with flowing the argon gas (99.99%) at a rate of 1.8 L/min through the system for 15 min, followed with heating the samples up to the targeted temperature of 1000 °C at a ramping rate of 20 °C/min, then held at 1000 °C for 1 h in a flowing argon (1.8 L/min) atmosphere at room pressure, and after that turned off the furnace and cooled down the samples below 200 °C, finally transferred the samples to a desiccator and cooled them down to ambient temperature before their weights were measured to record sample yields. Thermally treated KL, KL/FePs and KL/FeN samples were labeled as KL-1000, KL/FePs-1000 and KL/FeN-1000, respectively. The thermo-gravimetric (TG) analyses of KL, KL/FePs and KL/FeN samples were also performed on a 50H thermo-gravimetric analyzer (TA Instruments, New Castle, DE, USA). Specifically, ten milligrams of each of three precursors was heated from 50 to 1000 °C at a ramping rate of 20 °C/min in a flowing nitrogen (99.99%, 100 mL/min) atmosphere at room pressure.

### 2.4. Characterization

The FTIR-ATR spectra of raw KL, FePs, FeN, KL/FePs, and KL/FeN samples were recorded with the PerkinElmer FTIR spectrometer (PerkinElmer, Waltham, MA, USA) at a resolution of 2 cm^−1^ for 10 scans in 450 to 4000 cm^−1^ range. All powdered samples were pressed against the diamond crystal of the ATR device. Background spectrum obtained by scanning the air was subtracted from the sample spectrum before converted into transmittance units. The spectra were baseline-corrected by applied “Data Tune-up” using Spectrum^®^ Quant software (version 10.4.2, PerkinElmer, Waltham, MA, USA). X-ray diffraction (XRD) experiments of KL, FePs, KL/FePs, KL/FeN, KL-1000, KL/FePs-1000, and KL/FeN-1000 samples were carried out with an Ultima3 diffractometer (Rigaku, The Woodlands, TX, USA. CuKα radiation with *λ* = 1.5406 Å, scanning speed of 1°/min and step size of 0.05°). Samples for XRD experiments were ground in an agate mortar to fine powders prior to the test. The XRD patterns of KL/FePs-1000 and KL/FeN-1000 were fitted by pseudo-Voigt function in 2*θ* range of 41 to 48° to calculate the relative fractions of iron phases. The grain sizes of iron phases were calculated based on the fitted peaks using the Scherrer equation:

(1) $$L=\frac{0.9\lambda }{\sqrt{(B_{M}^{2}-B_{S}^{2}})\times \cos\theta }$$

where *λ* is the wavelength of target CuKα (1.5406 Å), *B_M_* is the full width at half maximum of fitted peak, *B_S_* is the full width at half maximum of the standard materials in radians (*B_S_* = 0.1° × π/180°), *θ* is the fitted peak centroid. The morphology of KL/FePs-1000 and KL/FeN-1000 samples were characterized by a JSM-6500F SEM (JEOL, Peabody, MA, USA) and a 2100-high-resulution TEM (JOEL, Peabody, MA, USA). The energy dispersive spectroscopy (EDS, Oxford Instruments, Scotts Valley, CA, USA) which coupled with SEM was used to analyze product elemental composition three independent measurements were conducted for each sample.

## 3. Results

### 3.1. Raw Materials and Precursors

#### 3.1.1. FTIR

**Figure 1:**
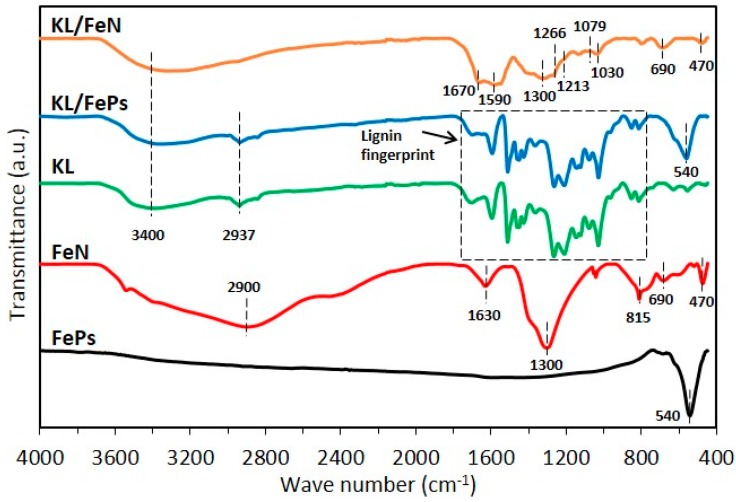
FTIR profiles of raw materials and KL/Fe mixtures.

Figure 1 illustrates FTIR spectra of KL, FePs, FeN, KL/FePs, and KL/FeN samples, and typical IR signals and their corresponded functional groups appeared in KL, FePs, and FeN samples are listed in Table 1. The FePs sample showed a Fe–O vibration adsorption peak at 540 cm^−1^ [18] because of the surface oxidation of FePs sample when exposed to air. The FeN sample had N–O (nitrate group, at 1300 and 815 cm^−1^) [19], Fe–O (476 cm^−1^) [20,21], and Fe–O–H (690 cm^−1^) [22], were attributes to. vibration adsorption peaks. KL/FePs samples had a very similar FTIR profile to KL samples, i.e., there was no changes occurred in lignin fingerprint region (800 to 1800 cm^−1^). This result indicates a simple mechanical blending of FePs and KL samples will not alter lignin macromolecular structure. In other words, there was only physical contact between iron and lignin, which evidenced a slight degree of contact between iron and lignin in KL/FePs sample. However, KL/FeN samples had a very different FTIR profile from KL samples. Specifically, the aliphatic C–O vibration (1030 and 1079 cm^−1^) and aromatic C–O stretching (1213 and 1266 cm^−1^) intensities of KL/FeN samples underwent great reductions. The C=O stretching bands of KL/FeN samples showed a red-shift from 1595 to 1590 cm^−1^ and from 1700 to 1670 cm^−1^. These changes suggest that the lignin macromolecular structure was altered through chelation of KL to iron ion (Fe^3+^) because of the high degree of contact between iron and lignin in KL/FeN samples.

#### 3.1.2. XRD

**Figure 2:**
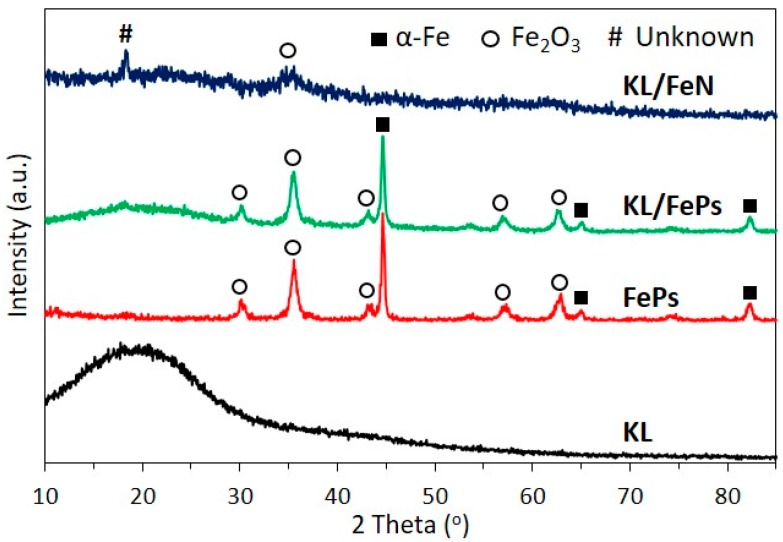
XRD patterns of KL, FePs, KL/FeN, and KL/FePs samples.

Figure 2 illustrates XRD patterns of KL, FePs, KL/FePs, and KL/FeN samples. The diffraction pattern of KL samples had a very broad peak around 21° only, corresponding to the amorphous structure of lignin. The diffraction pattern of FePs samples had iron oxides (Fe_2_O_3_) and body-centered cubic (α-Fe, pdf #03-065-2411) iron peaks. These iron peaks confirm that FePs were partly oxidized to iron oxides. KL/FePs samples had a very similar diffraction pattern to FePs samples, which confirms that lignin macromolecular structure was not altered because there was a low degree of contact between iron and lignin in KL/FePs samples. KL/FeN samples had only two very weak peaks at 18 and 35°, respectively. There were no iron nitrate related peaks (Figure S1) observed because of weak crystallization of iron nitrate and KL chelation KL to Fe^3+^. On the other hand, the missing peak also confirms the high degree of contact between iron and lignin in KL/FeN samples.

### 3.2. Thermally Treated Samples

#### 3.2.1. Thermal Treatment Yield and TG Analyses

**Figure 3:**
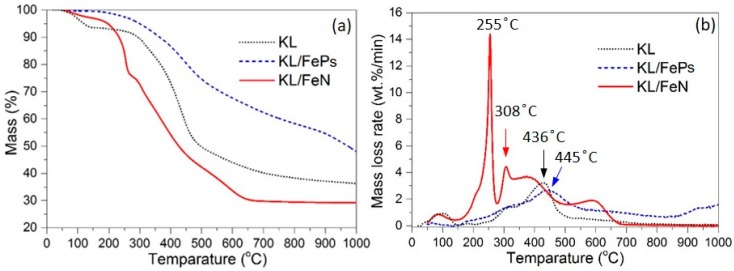
TG mass loss (**a**) and DTG (**b**) curves of KL, KL/FePs, and KL/FeN samples.

The thermal treatment yield of KL/FeP and KL/FeN were 53.68% (with its coefficient of variation of 0.6%) and 42.88% (1.0%), respectively. Figure 3 shows the TG mass loss and mass loss rate (DTG) curves of KL, KL/FePs, and KL/FeN samples and Table 2 summarizes the characteristic points appeared in the TG and DTG curves. The solid product yields of KL, KL/FePs, and KL/FeN samples were 36.4%, 48.0%, and 29.2%, respectively. The major weight loss of KL, KL/FePs, and KL/FeN samples (defined as a weight loss rate is greater than 1 wt %/min) occurred at the temperature range of 291 to 482, 275 to 689, and 182 to 636 °C, respectively. KL/FeN samples had two DTG peaks, i.e., the first peak centered at 255 °C mainly was related to the decomposition of nitrate compounds, and the second peak centered at 308 °C was because of lignin thermal decomposition.

Table 2 indicates that in general the presence of iron-based catalysts resulted in a decrease in the starting temperature of a major weight loss process (*T_start_*). The presence of catalysts helps accelerate the major weight loss process occurring in the lignin thermal decomposition, especially for KL/FeN samples because the macromolecular structure of lignin was modified by the chelation of Fe^3+^. KL/FePs samples had a weight loss of 14.4% which was much higher than the 0.6% of KL/FeN samples in the temperature range from 700 to 1000 °C. This is because some aromatic tars coked on FePs (Fe and Fe_2_O_3_) surfaces at the low temperature range (350 to 500 °C), followed by thermal creaking of aromatic tars to various volatile carbonaceous gases at high temperatures (700 to 1000 °C) [2,24].

#### 3.2.2. XRD

**Figure 4:**
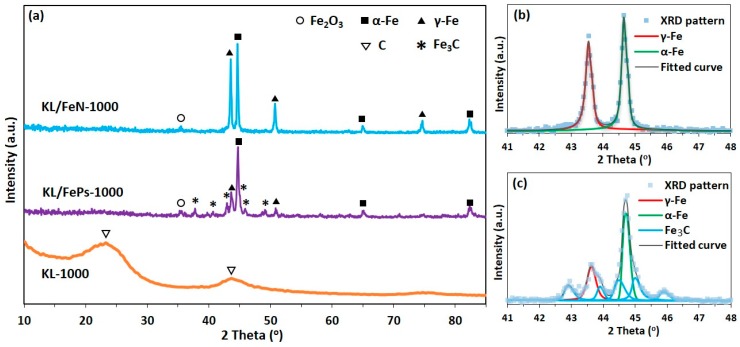
(**a**) XRD patterns of KL-1000, KL/FeN-1000, and KL/FePs-1000 samples; and fitted patterns of (**b**) KL/FeN-1000 and (**c**) KL/FePs-1000.

KL/FePs-1000 samples (Figure 4a) had three typical sharp peaks for α-Fe (pdf #03-065-2411), face-centered cubic (γ-Fe, pdf #98-000-0258), and Fe_3_C (pdf #03-065-2411) phases, respectively. KL/FeN1000 samples had metallic iron (α-Fe and γ-Fe) diffraction peaks, but no Fe_3_C diffractions detected. KL-1000 samples had only two broad humps indicating a disordered carbon structure. 

The relative fraction and grain size of each iron phase appeared in the XRD pattern was calculated using pseudo-Voigt function fitted XRD pattern in the 2*θ* range of 41 to 48°, and the fitted curves of KL/FePs-1000 and KL/FeN-1000 samples are shown in Figure 4b,c, respectively. KL/FeN-1000 samples contained 52% of α-Fe particles with their grain size averaging of 60 nm, and 48% of γ-Fe particles with their grain size averaging of 80 nm. KL/FePs-1000 samples contained 34% of α-Fe particles with their grain size averaging of 52 nm, 20% γ-Fe particles with their grain size averaging of 32 nm, and 46% of Fe_3_C particles with their grain size averaging of 48 nm. Only a very small Fe_2_O_3_ diffraction peak can be found at 35.6° on both XRD patterns of KL/FePs-1000 and KL/FeN-1000 samples because a very small amount of unprotected iron-nanoparticles was oxidized by air during the process of removing thermally treated samples from the furnace. These results suggested that the majority of metallic iron nanoparticles in thermally treated samples were protected from being oxidized by air.

#### 3.2.3. SEM and HRTEM

**Figure 5:**
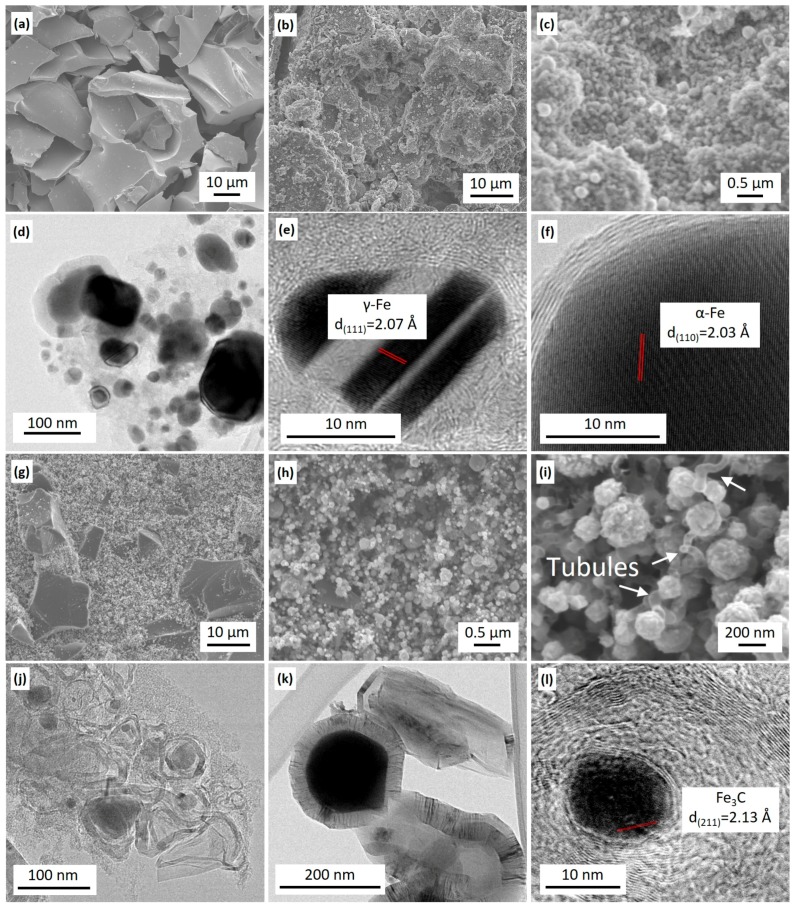
SEM images of KL-1000 samples (**a**) showing relative smooth surface, and KL/FeN-1000 samples (**b**) showing rugged surface structure with many nanoparticles (**c**); TEM images of KL/FeN1000 samples showing CEINs (**d**) with γ-Fe (**e**) and α-Fe cores (**f**); SEM images of KL/FePs-1000 samples showing complex surface structure (**g**) with many nanoparticles (**h**) and sinuous tubule (**i**); and TEM images of KL/FePs-1000 samples showing iron-based nanoparticles encapsulated by graphitic-carbon nanoribbons (**j**), carbon tubules (**k**), and a Fe_3_C core nanoparticle (**l**).

SEM images indicated that KL-1000 samples had a relative smooth surface (Figure 5a) and KL/FeN-1000 samples had a complex surface structure of many nanoparticles distributing on the rugged surface (Figure 5b,c). Elemental analyses (Figure S2a,b in Supplementary Materials) revealed that KL/FeN-1000 sample surfaces consisted of carbon (55 ± 5 wt %), iron (32 ± 5 wt %), oxygen (5 ± 3 wt %), sodium (5 ± 2 wt %), and sulfur (~1 wt %). This indicated that KL was carbonized and FeN was decomposed and reduced to iron nanoparticles covered by carbon. The sodium and sulfur were originally from pulping chemicals left in KL. The oxygen detected could be because sodium and unprotected iron nanoparticles reacted with air during the process of removing KL/FeN-1000 samples from the furnace [13].

TEM images of KL/FeN-1000 samples confirmed that iron nanoparticles were encapsulated by few layers graphitic-carbon (mostly 1–5 layers, in Figure 5d–f). These CEINs had lattice planes indexed to α-Fe (Figure 5e) and γ-Fe (Figure 5f), and these observations were in agreement with XRD results. These Fe phases (α-Fe and γ-Fe) were considered as active catalyst sites for the formation of graphitic-carbon layers around iron nanoparticles [4,6]. The majority of these CEINs were found embedded in amorphous carbon matrix. Thus, the formation of CEINs was attributed to solid carbon directly reacting with iron nanoparticles.

SEM image analyses indicated that KL/FePs-1000 samples had a complex and rough surface structure which was partially naked and smooth, and partially covered by nanoparticles (Figure 5g,h). The majority of nanoparticles were connected with sinuous tubules and ribbons (Figure 5i). Elemental analyses (Figure S2c,d) revealed that these surface nanoparticles consisted of carbon (11 ± 5 wt %), iron (75 ± 5 wt %), oxygen (13 ± 3 wt %), sodium (<1 wt %), and sulfur (<1 wt %), which corresponded to the graphitic-carbon nanostructures from iron graphitization of lignin. However, the naked surface consisted of 100% carbon (Figure S2e,f) that corresponded to amorphous carbon from thermal treatment of lignin without contact with iron. These observations also implied that iron nanoparticles were not uniformly distributed in KL matrix.

TEM images of KL/FePs-1000 samples revealed that iron nanoparticles were encapsulated in tangled graphitic-carbon nanoribbons (Figure 5j) and carbon tubules (Figure 5k). Graphitic-carbon nanoribbons and carbon tubules appeared only along the edge of amorphous carbon matrix. The nanoribbons had their thickness ranging from 5 to 20 nm and length of 10 nm. Carbon tubules had their inner diameters larger than 50 nm and length of a few hundred nanometers. Figure 5l indicated that iron nanoparticles had a lattice space of 2.13 Å corresponding to the (211) plane of Fe_3_C. The Fe_3_C phase was previously reported as the active catalyst for graphitic-carbon nanoribbon and carbon tubules formation in thermally treated wood chips promoted by iron-based catalysts, and it was believed that the formation of graphitic-carbon nanoribbons and carbon tubules was because iron-based catalysts directly reacted with solid carbon [13,15]. However, the formation of graphitic-carbon nanoribbon and carbon tubules observed in this study was based on carbonaceous gases such as methane reacting with surface iron-catalyst nanoparticles through a CVD process because these graphitic-carbon nanostructures only appeared on the amorphous carbon surface.

## 4. Discussion

During the thermal treatment of KL and iron-based precursors, the following reactions took place: (1) decomposition and transformation of lignin into amorphous carbon at 300 to 600 °C [2]; (2) transformation of iron catalysts to different phases of iron nanoparticles (α-Fe, γ-Fe, and/or Fe_3_C) at 600 to 1000 °C; (3) transformation of amorphous carbon that is in contact with iron nanoparticles to graphitic-carbon layers around iron nanoparticles to form CEINs at the temperature greater than 800 °C; (4) volatile gases (such as methane, carbon monoxide) from thermal decomposition of lignin reacting with iron nanoparticles to form various graphitic-carbon nanostructures.

**Figure 6:**
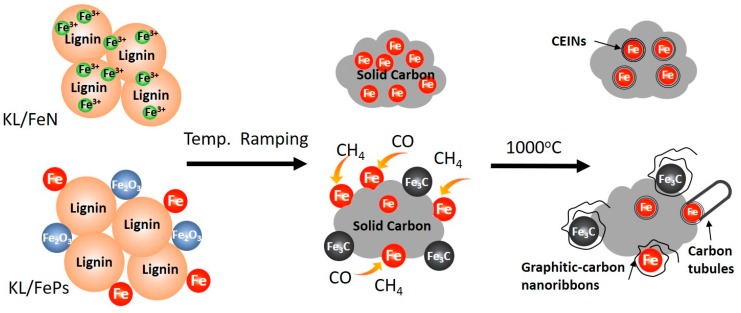
Formation mechanism of graphitic-carbon shells, graphitic-carbon nanoribbons, and carbon tubules around iron nanoparticles in two different catalyst systems.

Experimental results showed that CEINs synthesized from two different catalyst systems had different microstructures because of two different reaction processes illustrated in Figure 6. For KL/FeN precursor system, the initial degree of contact between iron and lignin was high because the solution-based FeN catalyst was used. Lignin was rapidly decomposed to amorphous carbon, and Fe^3+^ was converted to iron nanoparticles which were tightly trapped into amorphous carbon matrix as the temperature increased from 200 to 700 °C [1]. The amorphous carbon was transformed into graphitic-carbon shells encapsulating iron nanoparticles to form CEINs at 800–1000 °C.

In the case of KL/FePs precursor system, the initial degree of contact between iron (Fe and Fe_2_O_3_ nanoparticles) and lignin was low because the particle-based FePs catalyst was used. The majority of iron nanoparticles was not trapped in amorphous carbon matrix during the temperature ramping process. Therefore, limited amount of CEINs are formed through catalytic graphitization of amorphous carbon. On the other hand, the volatile tars could be absorbed on iron nanoparticle surfaces at 300 to 500 °C, followed by tars decomposed to carbonaceous gases (such as CO and CH_4_) at 600 to 1000 °C [2,24]. These CO and CH_4_ reacted with iron nanoparticles through a CVD process to grow carbon tubules and tangled graphitic-carbon nanoribbons around iron nanoparticles.

## 5. Conclusions

CEINs were synthesized through thermally treating KL using solution-based FeN and solid-based FePs as catalysts. The physical and chemical states of iron-based catalysts affected the formation process and morphology of as-synthesized CEINs. Both solid carbon and carbonaceous gases from KL thermal decomposition participated in the formation of CEINs. The formation of CEINs mainly resulted from solid carbon when solution-based FeN was used as the catalyst. The formation of CEINs mainly resulted from carbonaceous gases when solid-based FePs were used as the catalyst. 

## Figures and Tables

**Table 1:** Typical infrared signals and functional groups appeared in KL, FePs, and FeN samples [2,19,20,22,23].

Sample	Wave Number (cm^−1^)	Functional Groups
KL	~3400	O–H stretching
	~2937	C–H_x_ stretching
	1700–1730	C=O stretching (unconjugated)
	1595	aromatic skeletal vibration and C=O stretch
	1511	aromatic skeletal vibrations
	1455, 1425	–CH_3_ deformation
	1365	In-plane deformation vibration of phenolic
	1266, 1213	C–O stretching of guaiacyl ring
	1150	guaiacyl C–H deformation
	1079	C–O, secondary alcohols
	1030	C–O, primary alcohols
	967	–C–C–H and –HC=CH– deformation
	851, 812	C–H, aromatic
FeN	~2900, 1630	O–H stretching, non-dissociated water
	1300, 815	N–O, nitrate group
	690	Fe–O–H
	470	Fe–O
FePs	540	Fe–O
KL/FeN	1670	C=O stretching (unconjugated)
	1590	aromatic skeletal vibration and C=O stretch
	1300	N–O, nitrate group
	1266, 1213	C–O stretching of guaiacyl ring
	1079, 1030	aliphatic C–O vibration
	690	Fe–O–H
	470	Fe–O

**Table 2:** Characteristic points of TG and DTG curves of KL, KL/FePs, and KL/FeN samples.

Sample	Yield (%)	*T_start_* ^1^ (°C)	*T_max_* ^2^ (°C)	*T_end_* ^3^ (°C)	*dW*/*dT_max_* (wt %/min)	Δ*W*(*T_start_–T_end_*) ^4^ (%)	Δ*W*(*T*_700_*–T*_1000_) ^5^ (%)
KL	36.4	291	436	482	3.2	38.7	3.9
KL/FePs	48.0	275	445	689	2.7	33.6	14.4
KL/FeN	29.2	182	255, 308	636	14.4, 4.4	65.0	0.6

*T_star_*_t_^1^ is the starting temperature of a major weight loss process; *T_max_* ^2^ is the temperature at a maximum mass loss rate; *T_end_* ^3^ is the ending temperature of a major weight loss process; Δ*W*(*T_start_–T_end_*) ^4^ is the weight loss occurred between *T_start_* and *T_end_*; Δ*W*(*T*_700_*–T*_1000_) ^5^ is the weight loss occurred between 700 and 1000 °C.

## Supplementary Materials

The following are available online at www.mdpi.com/1996-1944/11/1/139/s1, Figure S1: XRD pattern of FeN (Iron(III) nitrate nonahydrate, Fe(NO_3_)_3_·9H_2_O). Figure S2: (a,c,e) SEM images and (b,d,f) the selected area SEM-EDS energy dispersive spectra of KL/FePs-1000 (a–d), and KL/FeN-1000 (e,f).
