# Supplementary material for: Effects of Physical and Chemical States of Iron-Based Catalysts on Formation of Carbon-Encapsulated Iron Nanoparticles from Kraft Lignin
Source: Materials (Basel). 2018 Jan 15;11(1):139. doi: 10.3390/ma11010139 (PMC5793637; doi:10.3390/ma11010139)
Supplement: Supplementary file 1 [file materials-11-00139-s001.pdf]

Effects of Physical and Chemical States of Iron-Based Catalysts on Formation of Carbon-Encapsulated Iron Nanoparticles from Kraft Lignin

Xuefeng Zhang [1], Qiangu Yan [1,*], Jinghao Li [2], Jilei Zhang [1,*] and Zhiyong Cai [2,*]

[1] Department of Sustainable Bioproducts, Mississippi State University, Mississippi State, MS 39762, USA
[2] U.S. Department of Agriculture, Forest Service, Forest Products Laboratory Madison, WI 53726, USA

Supplementary Materials

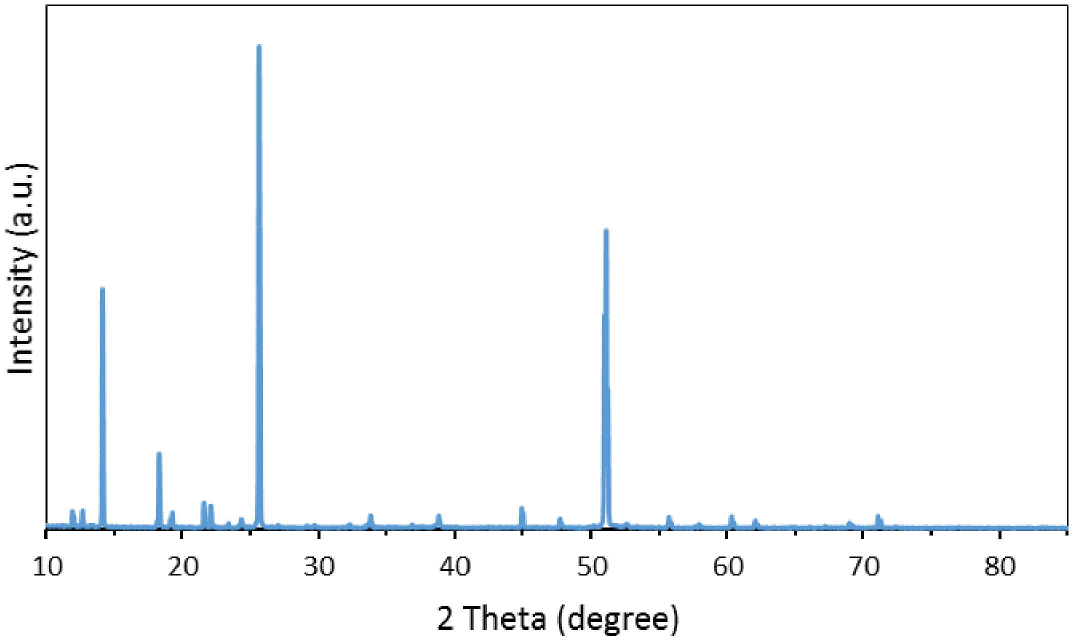

Figure S1. XRD pattern of FeN (Iron(III) nitrate nonahydrate, $Fe(NO_3)_3 \cdot 9H_2O$).

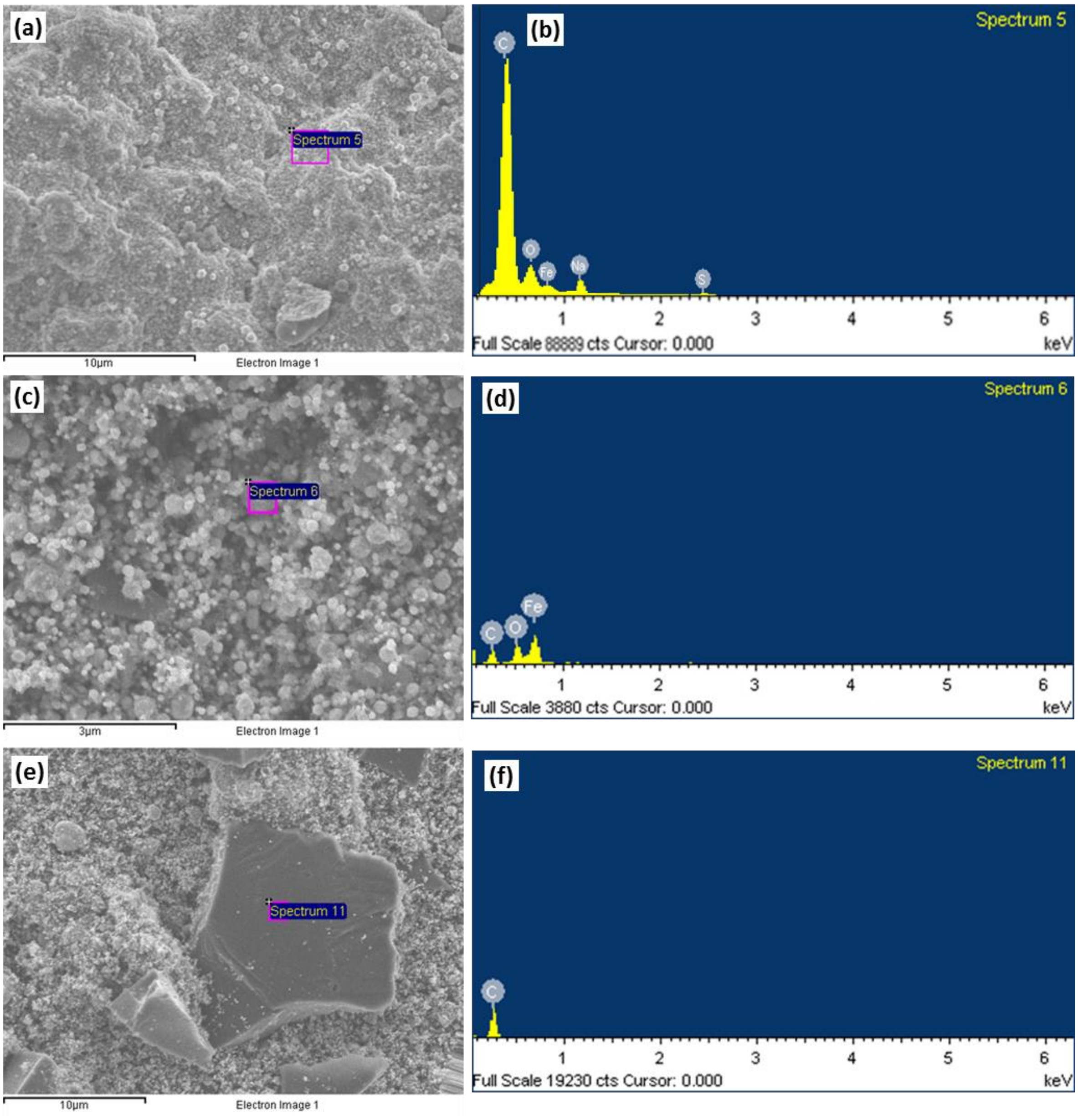

Figure S2. (**a**, **c**, and **e**) SEM images and (**b**, **d**, and **f**) the selected area SEM-EDS energy dispersive spectra of KL/FeN-1000 (**a** and **b**), surface nanoparticles of KL/FeP-1000 (c and d), and naked surface of KL/FeP-1000 (**e** and **f**).
